# Galectins as Master Regulators of Gastric Cancer Progression

**DOI:** 10.3390/cells14141090

**Published:** 2025-07-16

**Authors:** Bojan Stojanovic, Ivan Jovanovic, Milica Dimitrijevic Stojanovic, Bojan Milosevic, Marko Spasic, Bojana S. Stojanovic, Stefan Jakovljevic, Nenad Zornic, Danijela Jovanovic, Jelena Nesic, Milan Paunovic, Ivan Radosavljevic, Nenad Markovic, Mladen Pavlovic, Nikola Mirkovic

**Affiliations:** 1 Department of Surgery, Faculty of Medical Sciences, University of Kragujevac, 34000 Kragujevac, Serbia; 2 Center for Molecular Medicine and Stem Cell Research, Faculty of Medical Sciences, University of Kragujevac, 34000 Kragujevac, Serbia; 3 Department of Pathology, Faculty of Medical Sciences, University of Kragujevac, 34000 Kragujevac, Serbia; 4 Department of Pathophysiology, Faculty of Medical Sciences, University of Kragujevac, 34000 Kragujevac, Serbia; 5 Department of Internal Medicine, Faculty of Medical Sciences, University of Kragujevac, 34000 Kragujevac, Serbia

**Keywords:** gastric cancer, galectins, tumor microenvironment, epithelial–mesenchymal transition, immune evasion, chemoresistance, biomarkers, tumor progression

## Abstract

Gastric cancer remains a major global health challenge, largely due to its biological heterogeneity and limited treatment options for advanced stages. Among the numerous molecular players involved in its pathogenesis, galectins—β-galactoside-binding lectins—have emerged as key modulators of tumor behavior. These multifunctional proteins influence diverse processes including cell proliferation, invasion, immune evasion, stromal remodeling, and therapy resistance. Recent advances in experimental and clinical research have shed light on the complex roles of galectin family members—particularly Galectin-1, -3, and -9—in shaping the tumor microenvironment and driving disease progression. This review highlights the current understanding of galectin biology in gastric cancer, with emphasis on their structural characteristics, cellular localization, functional diversity, and translational relevance. By synthesizing insights from molecular studies and clinicopathological observations, we explore the potential of galectins as biomarkers and therapeutic targets in the evolving landscape of gastric cancer research.

## 1. Introduction

Gastric cancer (GC) remains a formidable health problem worldwide [1]. Although its global incidence has declined in recent decades, it still represents the fifth most frequently diagnosed malignancy and the third leading cause of cancer-related death [2,3]. More than one million new cases are recorded each year, yet their distribution is far from uniform: East-Asian countries carry a disproportionately high burden, whereas many Western nations experience comparatively lower rates [4,5]. This geographic heterogeneity underlines the complex interplay of environmental, infectious, and genetic factors in gastric carcinogenesis.

Well over 90% of primary malignant gastric tumors are adenocarcinomas [6]. Histologically, they are divided into two major Lauren categories [7]. Intestinal-type tumors form cohesive tubular or gland-like structures and often arise on a background of chronic gastritis and intestinal metaplasia [8]. Diffuse-type lesions, by contrast, consist of poorly adherent cells that infiltrate the gastric wall as scattered foci, frequently producing a desmoplastic, linitis-plastica-like phenotype [9]. From a surgical standpoint, the additional distinction between proximal (cardia/fundus) and distal (corpus/antrum) disease is clinically relevant, yet neither scheme alone reliably predicts therapeutic response or outcome [10].

Helicobacter pylori infection remains the single most powerful risk factor, particularly for distal tumors [11]. Other recognized contributors include high dietary salt and nitrate intake, smoking, atrophic gastritis, adenomatous polyps, and inherited cancer-prone syndromes [6]. For cancers of the proximal stomach, excess body weight, chronic gastro-esophageal reflux and Barrett’s metaplasia predominate [12]. On a molecular level, amplification of the proto-oncogene human epidermal growth factor receptor 2 (HER2) is detected in roughly one in six gastric adenocarcinomas, offering a rationale for targeted therapy in that subset [13].

Early-stage disease is typically silent or produces only vague dyspeptic complaints; consequently, a large proportion of patients present with stage III or IV tumors [14]. This diagnostic delay is a chief reason why, despite advances in endoscopy, imaging and systemic therapy, the overall 5-year survival rate still lingers below 30% in most regions [15]. Peritoneal seeding represents the most frequent metastatic route [16]. Approximately 14% of patients already harbor peritoneal deposits at initial diagnosis, and peritoneal relapse remains the commonest mode of failure even after apparently curative gastrectomy [17].

Given the disappointing survival figures and the limited predictive value of conventional clinicopathological staging, there is an urgent need to elucidate the molecular circuitry that governs invasion and spread. Identifying robust biomarkers for early detection, risk stratification and therapeutic guidance would not only improve individual patient management but also inform the design of future targeted or immunomodulatory strategies—settings in which galectins have recently emerged as promising protagonists.

Although galectins drive tumor progression in many solid malignancies, comparative data show that their regulatory landscape in gastric cancer is distinctive enough to merit a dedicated synthesis. In gastric tumors, cancer-associated fibroblasts—not the malignant epithelium—are the principal source of Galectin-1, establishing a stromal signaling loop that promotes invasion and suppresses antitumor immunity [18]. By contrast, Galectin-1 is synthesized mainly inside tumor cells in hepatocellular, colorectal, and breast carcinomas, where it supports proliferation, angiogenesis, and metastatic spread [19,20,21]. Prognostically, high Galectin-9 expression is associated with longer survival in gastric cancer, yet marks T-cell exhaustion and poorer outcomes in non-small-cell lung cancer, underscoring the context-dependent nature of this lectin’s immune regulation [22,23]. Gastric cancer cells also release Galectin-3-rich exosomes that remodel the peritoneum into a fibrotic, CXCL12-laden niche, thereby favoring trans-coelomic dissemination—the dominant metastatic route of this disease [24]. Finally, chronic *Helicobacter pylori* gastritis alters mucin glycosylation and increases β-galactoside structures, broadening the reach of galectin signaling to a degree not reported in other digestive or gynecologicaltumors [25]. Taken together, these tumor-, stromal-, and microbe-driven features identify galectins as central regulators with a gastric-specific signature and provide the rationale for the focused overview offered in this review.

## 2. Galectins: An Introduction to the β-Galactoside Lectin Family

Among the many carbohydrate-binding proteins found in humans, galectins form a distinctive subgroup recognized for their affinity toward β-galactoside-containing glycans [26]. Current comparative-genomic analyses suggest that mammals possess at least 22 galectin genes; 16of these are expressed in human tissues [27]. Their chronological nomenclature—from galectin-1, first isolated in 1975, to the most recently annotated variants—reflects the order of discovery rather than functional hierarchy [28].

**Figure 1:**
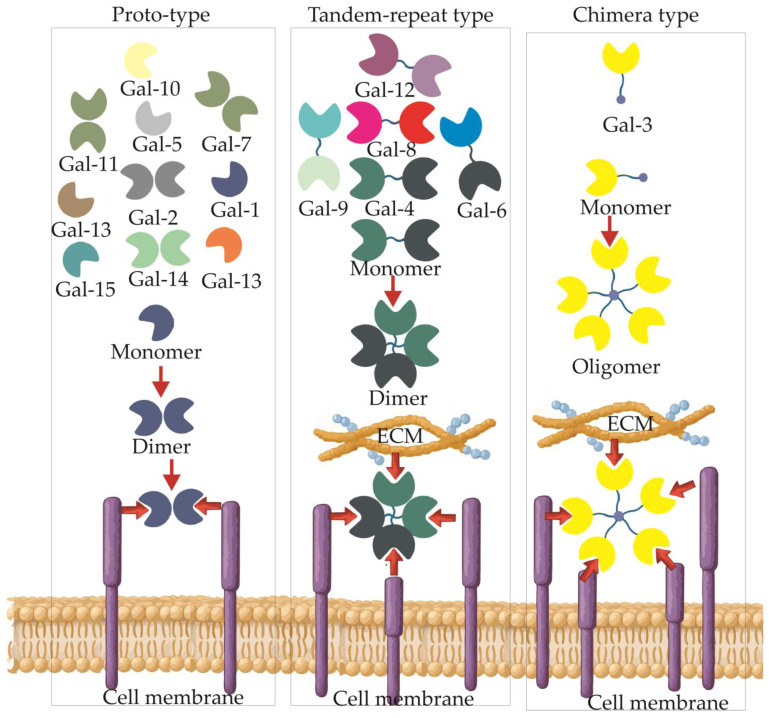
Structural classification of human galectins. Galectins are grouped into three main types based on the organization of their CRDs. Proto-type galectins (left) contain a single CRD and form non-covalent homodimers (e.g., Gal-1, -2, -7). Tandem-repeat type galectins (middle) harbor two distinct CRDs linked by a peptide spacer within the same polypeptide (e.g., Gal-4, -8, -9), enabling bivalent binding. Chimera-type galectin (right), represented solely by Gal-3, contains a single CRD and a unique N-terminal domain allowing oligomerization. All galectin types interact with glycosylated receptors at the cell membrane, promoting lattice formation and downstream signaling.

Every galectin harbors one or two highly conserved carbohydrate-recognition domains (CRDs) of roughly 130–135 amino acids [29]. Based on the number and disposition of these CRDs, the family is subdivided into three structural categories (Figure 1) [29]:

- Proto-type (e.g., galectin-1 [Gal-1], Gal-2, Gal-7): single CRD that operates either as a monomer or a non-covalent homodimer.
- Tandem-repeat type (e.g., Gal-4, -8, -9): two distinct CRDs bridged by a flexible linker within one polypeptide chain.
- Chimera type (Gal-3): a solitary CRD at the C-terminus attached to an N-terminal domain rich in proline, glycine and tyrosine, conferring remarkable multimerization capacity.

This modular organization endows galectins with the versatility to form lattices with glycoproteins and thereby fine-tune cell-surface receptor clustering, endocytosis, and signaling [30].

Galectins are not confined to vertebrates; homologues occur throughout the animal kingdom and even in some microbes, underscoring their ancient evolutionary origin [31]. In humans, basal expression is almost ubiquitous, yet levels fluctuate markedly in response to developmental cues, inflammation, hypoxia, and malignant transformation [32].

Galectins operate at multiple levels of the immune response, acting as carbohydrate-dependent sentinels that sense pathogenic and damage-associated glycans while also shaping intercellular communication [33]. In innate immunity, galectins bind to β-galactoside-rich ligands on neutrophils and macrophages. This interaction enhances phagocytosis, promotes the oxidative burst, and stimulates early cytokine release [34,35]. Within phagosomes, certain galectin family members—particularly galectin-3—stabilize the vacuolar membrane. They also fine-tune inflammasome activation, ensuring that microbial clearance is accompanied by a controlled inflammatory response [34,35]. Within the adaptive compartment galectins function as “glyco-checkpoints”: by selectively triggering apoptosis in pro-inflammatory T-helper subsets or by reinforcing the survival of regulatory T cells, they recalibrate the balance between effector reactivity and tolerance [33,36,37]. Engagement of receptors such as TIM-3 by galectin-9 further tempers hyperactivated or exhausted T lymphocytes, leading to increased production of immunosuppressive mediators like interleukin-10 [38]. Beyond direct effects on leukocytes, galectins modulate the surrounding cytokine milieu by favoring antiinflammatory signals, dampening nuclear factor κB and JAK/STAT pathways, and reorganizing chemokine gradients through the formation of glycan lattices on endothelial and stromal surfaces [39]. Because similar glycan signatures decorate immune, stromal and malignant cells alike, these lectins exert comparable regulatory influence in autoimmunity, chronic infection, transplantation, and diverse solid or hematologicaltumors, making them attractive yet complex therapeutic targets.

## 3. Galectin-1 in Gastric Cancer: A Multifaceted Modulator of Tumor Progression

Galectin-1 (Gal-1) plays a critical role in gastric cancer by regulating immune evasion, matrix remodeling, angiogenesis, and epithelial–mesenchymal transition. Its upregulation in both tumor cells and stromal compartments, especially in cancer-associated fibroblasts, contributes to tumor growth, invasiveness, and therapy resistance. Understanding its mechanisms of action offers valuable insight into gastric cancer pathogenesis and potential therapeutic targets.

### 3.1. Galectin-1: Structure, Function, and Its Pivotal Role in Tumor Biology

Galectin-1 was the first discovered and isolated member of the galectin family [40]. It is encoded by *LGALS1* and is synthesized as a 14-kDa polypeptide that homodimerizes through non-covalent forces, thereby creating two spatially opposed β-galactoside-binding pockets [41]. Galectin-1 lacks a classical signal peptide and is therefore exported via an unconventional secretory route. After translation on cytoplasmic ribosomes, it translocates to the inner leaflet of the plasma membrane. From there, it is released into the extracellular space. In this environment, its multivalent nature allows cross-linking of glycosylated receptors and extracellular matrix components [42,43,44].

Extracellular Gal-1 is a potent immunomodulator [45]. By binding to glycan ligands on activated T cells, Gal-1 induces Fas-dependent apoptosis, suppresses the release of pro-inflammatory cytokines, promotes the expansion of regulatory T-cell subsets, and inhibits the maturation of dendritic cells [37,44,46]. Collectively, these effects contribute to the establishment of an immune-privileged microenvironment that can be exploited by tumors to evade host immune surveillance.

Within the cytoplasm, Gal-1 interacts with the rat sarcoma viral oncogene homolog (RAS)-binding domain of rapidly accelerated fibrosarcoma (RAF) kinases, stabilizing Harvey rat sarcoma viral oncogene homolog (H-RAS) nanoclusters and enhancing RAF–mitogen-activated protein kinase kinase (MEK)–extracellular signal-regulated kinase (ERK) signaling [47]. In the nucleus, it interacts with transcriptional regulators such as forkhead box P3 (FOXP3) and gem-associated protein 4 (Gemin4), providing an additional layer of control over gene expression programs that influence proliferation, survival, and epithelial–mesenchymal plasticity [48].

At the cell surface, Gal-1 organizes ‘lattices’ by bridging integrins, laminin, fibronectin, and other basement-membrane glycoproteins [27]. Depending on local glycan density, this scaffold can either tighten or loosen cell–matrix adhesion. In one scenario, Gal-1 stabilizes tumor–stroma contacts to facilitate invasion; in another, it competes with matrix ligands, promoting detachment of cancer cells that subsequently seed secondary sites [49,50].

Galectin-1 is now recognized as a multilayered orchestrator of tumor progression, acting through both cellular signaling and matrix-modifying routes. Several stimuli regulate LGALS1 transcription. Hypoxia, oncogenic RAS signaling, and inflammatory cytokines act through hypoxia-inducible factor 1 (HIF-1) and activator protein 1 (AP-1) binding motifs. These signals strongly induce Gal-1 expression in malignant epithelial cells, cancer-associated fibroblasts (CAFs), endothelial cells, and infiltrating myeloid populations [51].

Extracellular dimers engage β-galactoside-rich glycans on activated T cells, triggering c-Jun N-terminal kinase/activator protein 1 (JNK/AP-1)-dependent apoptosis and sharply reducing intra-tumoral cluster of differentiation 8-positive (CD8^+^) infiltrates [52,53,54]. Parallel binding to dendritic cells arrests their maturation, while interaction with naive CD4^+^ subsets promotes FOXP3^+^ T-regulatory differentiation [55,56]. The net result is a tolerogenic microenvironment that blunts cytotoxic immunity and facilitates tumor escape [56].

Gal-1 cooperates with transforming growth factor-beta (TGF-β) to convert resident fibroblasts into contractile CAFs that secrete additional Gal-1, collagen, and matrix metalloproteinases [57]. Simultaneously, Gal-1 ligates laminin- and fibronectin-bound integrins on endothelial cells, potentiating H-RAS/Raf/MEK/ERK signaling, endothelial proliferation, and sprouting [58]. These effects can sustain vascular expansion even when vascular endothelial growth factor (VEGF) pathways are pharmacologically blocked, underscoring Gal-1’s capacity to bypass antiangiogenic therapy [59].

By clustering β1-integrins and loosening cell–matrix contacts, Gal-1 lowers the mechanical threshold for epithelial detachment, facilitates epithelial–mesenchymal transition via Hedgehog (Hh)–glioma-associated oncogene homolog 1 (Gli1) activation, and enhances protease-mediated matrix degradation [60].

Over-expression of Gal-1 in cancer epithelium and stroma correlates with greater tumor mass, higher metastatic burden, and shorter patient survival in breast, prostate, and laryngeal cancers [61,62,63]. Small-molecule inhibitors, neutralizing antibodies, and glycan mimetics that interfere with Gal-1 lattice formation are currently being investigated as adjuvant therapeutic strategies aimed at restoring antitumor immunity, inhibiting angiogenesis, and restricting tumor dissemination [64].

### 3.2. Galectin-1 Expression in Normal Gastric Mucosa and Precancerous Conditions

In the healthy gastric mucosa, Gal-1 expression is typically confined to the stromal compartment, with little to no detectable presence in the epithelial cells [65]. Immunohistochemical studies have demonstrated a lack of Gal-1 staining within the epithelial layer under physiological conditions, suggesting that this lectin does not participate in the baseline homeostatic functions of the gastric epithelium [65].

However, this expression profile undergoes a marked shift during the transition from normal to premalignant states. The intestinal-type variant of gastric cancer, which is the most prevalent histological subtype, is thought to arise through a well-defined cascade of histopathological changes—beginning with Helicobacter pylori-induced superficial gastritis, progressing to chronic atrophic gastritis, intestinal metaplasia (IM), dysplasia, and ultimately carcinoma. Each of these stages reflects a stepwise accumulation of molecular and cellular alterations that contribute to malignant transformation [66].

Among these precancerous conditions, IM represents a critical juncture in gastric carcinogenesis [67]. Characterized by the replacement of native gastric epithelial cells with those resembling intestinal lineage, IM is observed in over 80% of patients diagnosed with intestinal-type adenocarcinoma [67,68]. At the molecular level, a notable upregulation of *LGALS1*—the gene encoding Gal-1—has been documented in gastric tissues with IM, particularly among female patients [65]. Immunohistochemical analysis of IM lesions reveals intense Gal-1 staining in both the epithelial cytoplasm and nuclei, as well as in the underlying stroma [65]. The marked increase in Gal-1 expression within these tissues—especially compared to normal mucosa—indicates its possible contribution to epithelial transformation and stromal remodeling, both of which are pivotal events in early tumor development.

Chronic gastritis, another key precursor in the pathway to intestinal-type GC, is similarly associated with elevated Gal-1 levels. Inflammatory microenvironments promote Gal-1 expression, which is detected in both epithelial and stromal regions of chronic gastritis [65,69]. The incremental rise of Gal-1 from chronic inflammation to IM and dysplasia dovetails with its known roles in immunomodulation, fibroblast activation, and extracellular-matrix remodeling. Elevated stromal levels can dampen mucosal cytotoxic T-cell surveillance, whereas epithelial Gal-1 may lower inter-cellular adhesion and prime cells for migration. Collectively, these changes provide a permissive niche in which more than 80% of intestinal-type carcinomas ultimately arise.

### 3.3. Galectin-1 Expression Patterns in Gastric Adenocarcinoma and Clinical Associations

Multiple studies have demonstrated that Gal-1 is markedly overexpressed in gastric adenocarcinoma tissues compared to adjacent non-malignant mucosa [18,57,65,69,70]. However, the distribution of Gal-1 within the tumor microenvironment appears to be spatially and cellularly compartmentalized. High levels of Gal-1 are consistently observed within the tumor-associated stromal cells, whereas its presence in the epithelial tumor cells is generally less pronounced [57,70,71,72,73].

The intensity of Gal-1 immunostaining within the stromal compartment has shown significant correlations with a range of pathological features, including tumor location, depth of invasion, histological differentiation, tumor, node, metastasis (TNM) stage, and presence of lymph node metastases [57,70,71,72,73]. Additionally, higher Gal-1 expression has been linked to adverse features such as perineural infiltration, serosal involvement, and increased tumor size. Clinically, patients exhibiting elevated stromal Gal-1 expression tend to have a significantly reduced five-year survival rate in comparison to those with low stromal Gal-1 levels, reinforcing its potential prognostic value [57,70,73].

Although the expression of Gal-1 in epithelial tumor cells of intestinal-type gastric cancer is typically low, a distinct pattern has emerged in signet ring cell carcinoma (SRCC), a diffuse subtype of gastric cancer [74]. In SRCC, Gal-1 expression is frequently observed within tumor cells, predominantly localized to the cytoplasm. This differential expression likely reflects the divergent oncogenic mechanisms underlying SRCC as compared to the gland-forming intestinal-type adenocarcinomas, suggesting that Gal-1 may play subtype-specific roles in gastric cancer pathogenesis.

Nonetheless, not all investigations have confirmed a consistent overexpression of Gal-1 in gastric malignancy. For instance, a study by Bektas et al. [71] reported a lack of Gal-1 upregulation in a substantial number of GC specimens, including areas of metaplasia and dysplasia. Such discrepancies may stem from methodological differences, tumor heterogeneity, or the dynamic regulation of Gal-1 in response to microenvironmental cues.

In addition to tissue-based analyses, circulating autoantibodies against Gal-1 (serum Gal-1 antibodies, s-GAL-1-Abs) have also been investigated as potential biomarkers. A study identified the presence of these antibodies in approximately 6.9% of patients with GC, with higher seropositivity observed in individuals with advanced-stage disease (stage III/IV) compared to those in early stages (0–II) [75]. These findings align with the broader literature suggesting that serum autoantibodies against tumor-associated antigens may serve as early indicators of malignancy and might reflect the biological behavior of the tumor more reliably than conventional serum tumor markers [76].

### 3.4. Galectin-1 in Cancer-Associated Fibroblasts: A Driver of Stromal Activation in Gastric Cancer

While tumor cells are the hallmark of cancer, the tumor microenvironment (TME)—comprising stromal cells, extracellular matrix (ECM), and signaling molecules—plays a decisive role in tumor progression [77]. Among stromal elements, cancer-associated fibroblasts (CAFs) are especially prominent. Originating from resident fibroblasts under the influence of tumor-derived signals, particularly transforming growth factor beta 1 (TGF-β1), CAFs gain a myofibroblast-like phenotype and actively support tumor growth, invasion, and metastasis [78].

Galectin-1, encoded by the *LGALS1* gene, has emerged as a critical effector molecule within CAF populations in GC [79]. Comparative analyses reveal markedly elevated Gal-1 expression in CAFs isolated from GC tissues versus normal fibroblasts (NFs) from adjacent mucosa [18,73]. This upregulation appears to be driven in part by TGF-β1, a cytokine abundantly present in the gastric TME [57]. TGF-β1 promotes Gal-1 transcription via mothers against decapentaplegic homolog 2 (Smad2) signaling and induces phenotypic transformation of NFs into alpha-smooth muscle actin (α-SMA)-positive CAFs [57]. In this setting, Gal-1 functions not only as a marker of fibroblast activation but also as an active mediator of stromal remodeling and tumor support.

Clinically, stromal Gal-1 expression has been correlated with key indicators of aggressive disease [57,70,71,72,73]. High Gal-1 levels in CAFs co-localize with β1 integrin, a cell adhesion molecule involved in migration and ECM interactions [18]. Their combined overexpression is significantly associated with deeper mural invasion, nodal metastasis, and higher TNM stages [18]. CAF-derived Gal-1 exerts multifaceted effects that reinforce tumor progression in gastric cancer. Through paracrine secretion of cytokines and matrix-remodeling enzymes, Gal-1-enriched CAFs enhance the proliferative, invasive, and survival capacities of malignant epithelial cells. Its accumulation in the tumor stroma, particularly in conjunction with β1-integrin signaling, is associated with enhanced extracellular matrix remodeling and deeper tumor infiltration. Moreover, Gal-1-mediated engagement of glycan structures on infiltrating T cells contributes to localized immunosuppression, further enabling tumor evasion and metastatic progression. These synergistic actions position CAF-derived Gal-1 as a critical modulator of the tumor microenvironment and a central orchestrator of gastric cancer aggressiveness.

### 3.5. Molecular Mechanisms of Galectin-1-Mediated Tumor Progression in Gastric Cancer

Galectin-1 exerts multiple tumor-promoting functions in GC, actively contributing to the malignant phenotype through regulation of proliferation, migration, invasion, and survival of tumor cells [80]. In vitro experiments with GC cell lines have provided mechanistic insight into these processes, underscoring Gal-1 as a pivotal effector in the intracellular and extracellular signaling landscape. Functional studies utilizing *LGALS1* knockdown in the HGC-27 gastric carcinoma cell line demonstrated that silencing Gal-1 significantly suppressed cellular proliferation, as well as migratory and invasive capacities [81]. These findings confirm that endogenous Gal-1 expression is essential for maintaining the aggressive phenotype of GC cells. Conversely, exogenous Gal-1 stimulation has been shown to enhance these tumorigenic traits, further affirming its role in driving GC progression [80].

Recent investigations have identified the glucose-regulated protein 78 (GRP78)—an endoplasmic-reticulum-resident oncoprotein—as a direct binding partner of Gal-1 [81]. GRP78 plays a vital role in the unfolded protein response and has been implicated in promoting cancer cell survival under stress conditions [82]. The Gal-1/GRP78 interaction was found to potentiate the malignant behavior of GC cells, promoting their proliferation and metastatic capacity [81].

In parallel, the neuropilin-1 (NRP-1)/jun proto-oncogene (c-JUN)/WEE1 G2 checkpoint kinase (Wee1) signaling axis has emerged as a critical downstream effector of Gal-1. Neuropilin-1, a transmembrane co-receptor involved in angiogenesis and cell migration, is activated upon binding with Gal-1 [80]. In GC cells, Gal-1 stimulation enhances the expression of NRP-1, which in turn activates the transcription factor c-JUN, a key component of the AP-1 complex [80]. This leads to the transcriptional upregulation of *Wee1*, a kinase that halts cell cycle progression at the G2/M checkpoint by inhibiting cyclin-dependent kinase 1 (CDK1) [80]. This Gal-1-driven activation of the NRP-1/c-JUN/Wee1 pathway fosters cell cycle arrest in response to DNA damage.

Moreover, Gal-1 has been implicated in the activation of two key oncogenic signaling cascades—MAPK and PI3K/AKT pathways—upon intracellular accumulation [83]. Activation of these pathways not only supports proliferative and antiapoptotic signaling but also endows GC cells with stem cell-like characteristics, including enhanced self-renewal and differentiation potential [83].

Interestingly, the tumor suppressor gene *RASSF1A* has been reported to positively regulate *LGALS1* expression at the mRNA level, while concurrently inhibiting the nuclear factor kappa-light-chain-enhancer of the activated B cells (NF-κB) pathway [84]. This paradoxical finding implies a complex regulatory role for Gal-1, potentially balancing proliferative signaling with cell cycle control under specific molecular contexts.

### 3.6. Galectin-1 in Metastatic Progression of Gastric Cancer: Molecular Drivers and Clinical Relevance

Most patients with GC are diagnosed at advanced stages, frequently presenting with local invasion or distant metastases [85]. Metastatic dissemination remains the leading cause of mortality in GC and involves a series of complex, multistep biological processes, including epithelial–mesenchymal transition (EMT), degradation of ECM, intravasation, survival in circulation, and colonization of distant organs [86]. A crucial early step in metastatic progression is the remodeling of the ECM, which provides both structural support and biochemical signals to cancer cells [87]. Integrins—transmembrane adhesion receptors—play a pivotal role in this context. By linking the ECM to the actin cytoskeleton, integrins enable cancer cells to sense and respond to environmental cues such as growth factors, chemokines, and cytokines secreted by stromal components, including CAFs and inflammatory cells [88]. Among integrins, β1 integrin has emerged as a key mediator of invasion and metastasis in GC [18].

Galectin-1 has been shown to promote the invasiveness of GC cells by enhancing the functional output of the integrin β1 signaling axis [18]. The Gal-1/integrin β1 interaction facilitates adhesion dynamics, cytoskeletal reorganization, and ECM degradation, ultimately supporting increased migratory and invasive capacities of GC cells [18]. This axis exemplifies the molecular cross-talk between tumor cells and stromal components that is essential for effective dissemination.

In addition to integrin-mediated effects, Gal-1 also regulates the expression of key intracellular effectors involved in tumor progression. Recent studies have identified the non-SMC condensin I complex subunit G (NCAPG) as a downstream target of Gal-1 [89]. NCAPG is a structural protein critical for chromosomal segregation and mitotic stability [90]. Gal-1 enhances NCAPG expression at both the mRNA and protein levels in GC cells [89]. Functional analyses suggest that NCAPG overexpression promotes antiapoptotic signaling, possibly through activation of the Wnt/β-catenin pathway—a signaling cascade intimately linked with EMT and cancer stemness [89]. These effects contribute to enhanced tumor cell survival, proliferation, and metastatic potential.

Furthermore, Gal-1 has been implicated in the most common and therapeutically challenging form of GC dissemination—peritoneal metastasis [91]. This form of spread, often detected postoperatively, severely limits therapeutic options and significantly worsens prognosis [92]. Gal-1 expression in primary GC tissues has been shown to positively correlate with increased peritoneal collagen deposition, particularly types I and III, and fibronectin (FN1), which collectively contribute to peritoneal fibrosis. In both human samples and animal models, elevated *LGALS1* expression was associated with thicker collagen layers in the peritoneum and increased incidence of metastatic nodules [91]. Mechanistically, Gal-1-driven peritoneal fibrosis creates a pro-metastatic niche, facilitating the attachment, survival, and outgrowth of disseminated tumor cells on the peritoneal surface.

### 3.7. Galectin-1 as a Regulator of EMT and Invasion in Gastric Cancer

Metastatic progression in gastric cancerinvolves epithelial–mesenchymal transition, a key mechanism whereby epithelial tumor cells lose their polarity and adhesion properties, acquire mesenchymal traits, and gain invasive and migratory capacities [93]. EMT is characterized by the downregulation of epithelial markers such as E-cadherin and the upregulation of mesenchymal markers like vimentin, facilitating stromal infiltration and metastatic dissemination. Multiple signaling pathways contribute to EMT, including TGF-β/Smad, Wnt/β-catenin, PI3K/AKT/mTOR, and Hedgehog (Hh) signaling [93].

Galectin-1 has emerged as a potent inducer of EMT in GC [73]. Elevated Gal-1 expression is positively correlated with vimentin and glioma-associated oncogene homolog 1 (Gli1)—a key transcriptional effector of the Hh pathway—and inversely associated with E-cadherin levels in GC tissues [73]. Mechanistically, Gal-1 promotes GC cell invasion and migration by activating both classical TGF-β/Smad signaling and non-classical Hh/Gli1 pathways [79]. In vitro, Gal-1 stimulation upregulates Gli1 and vimentin expression while downregulating E-cadherin, driving EMT and enhancing malignancy [79].

Importantly, β1 integrin has been identified as a critical mediator of Gal-1-induced EMT [73]. It facilitates Gal-1-driven Gli1 expression and subsequent EMT activation. Silencing β1 integrin in GC cells inhibits Gli1 upregulation and abrogates the pro-invasive effects of CAF-derived Gal-1 [73]. This Gal-1/β1 integrin/Gli1 axis represents a central pathway through which Gal-1 orchestrates tumor cell plasticity, migration, and metastatic potential.

### 3.8. Galectin-1 and Vasculogenic Mimicry in Gastric Cancer

Vasculogenic mimicry (VM) is a phenomenon whereby aggressive tumor cells form vascular-like networks that facilitate blood supply independently of classical angiogenesis [94]. Initially described in melanoma, VM arises when endothelial-driven vessel formation cannot meet the metabolic demands of rapidly growing tumors [95]. Instead, tumor cells themselves organize into non-endothelial vascular channels that mimic normal vasculature, allowing direct perfusion and connection with the host circulatory system [96]. These pseudo-vessels lack endothelial lining but contain a basement membrane and are composed entirely of malignant cells. In gastric cancer, VM has been detected in primary tumors and is associated with increased metastasis, poor postoperative prognosis, and resistance to antiangiogenic therapies [97].

Recent evidence indicates that Gal-1 is a key promoter of VM in GC [97]. Acting both extracellularly and intracellularly, Gal-1 supports the formation of these non-endothelial networks through multiple mechanisms. It enhances EMT, a prerequisite for the cellular plasticity required to form VM structures. Gal-1-induced EMT is mediated in part via activation of the Hedgehog signaling pathway, specifically through upregulation of its terminal effector Gli1. In GC tissues, Gal-1 expression positively correlates with Gli1 activity, linking this pathway to VM development [97].

Moreover, Gal-1 can activate Hh/Gli1 signaling via Ras-dependent mechanisms [98]. As an intracellular scaffold protein, Gal-1 stabilizes H-Ras nanoclusters, potentiating downstream Ras signaling, which in turn is known to drive Sonic Hedgehog (SHh) expression. Through this cascade—Ras/SHh/Hh-Gli—Gal-1 contributes to the reprogramming of tumor cells toward a VM phenotype [98].

### 3.9. Galectin-1 and Chemotherapy Resistance in Gastric Cancer

Platinum-based chemotherapy remains a cornerstone in the treatment of GC [99]. However, both intrinsic and acquired resistance significantly compromise its long-term efficacy [99]. The mechanisms underlying chemoresistance in GC are multifactorial and include impaired apoptotic signaling, dysregulation of cell cycle checkpoints, enhanced autophagy, increased DNA repair capacity, and activation of EMT and cancer stem cell (CSC) pathways [100]. Furthermore, stromal elements of the tumor microenvironment, notably CAFs, also contribute to therapeutic resistance.

Galectin-1 has been implicated in modulating resistance to cisplatin-based chemotherapy in GC [80]. In vitro studies have shown that elevated intracellular Gal-1 levels reduce GC cell sensitivity to cisplatin, suggesting a direct role in drug resistance [80]. Mechanistically, Gal-1 activates the MAPK and PI3K/AKT signaling pathways, which are associated with enhanced cell survival and stemness features. These Gal-1-induced stem-like cancer cells often display reduced endocytic capacity, limiting drug uptake and thereby facilitating chemoresistance [83].

**Figure 2:**
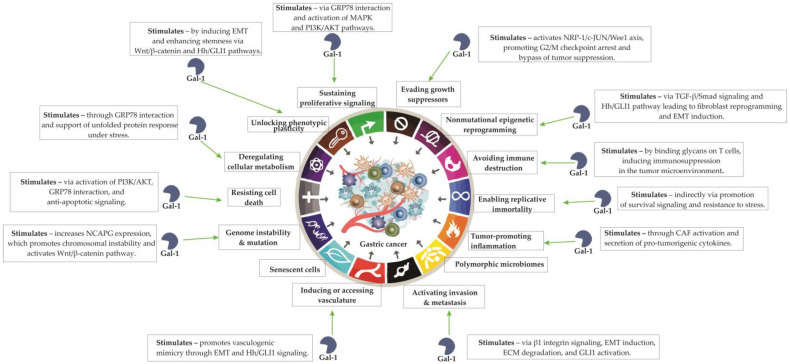
Galectin-1 and its role in promoting the hallmarks of gastric cancer. Galectin-1 promotes numerous hallmarks of cancer in gastric adenocarcinoma through diverse mechanisms. It sustains proliferative signaling via activation of MAPK and PI3K/AKT pathways; enables evasion of growth suppressors by activating the NRP-1/c-JUN/Wee1 axis; supports non-mutational epigenetic reprogramming and EMT induction through TGF-β/Smad and Hh/GLI1 pathways; facilitates immune evasion by inducing T cell suppression; enables replicative immortality via prosurvival signaling; promotes inflammation through activation of cancer-associated fibroblasts (CAFs) and pro-tumorigenic cytokines; enhances invasion and metastasis via β1 integrin signaling, ECM remodeling, and EMT; supports vascular mimicry via EMT and GLI1; increases genome instability through NCAPG upregulation and Wnt/β-catenin activation; resists cell death by PI3K/AKT signaling and GRP78 interaction; deregulates cellular metabolism by sustaining the unfolded protein response; and unlocks phenotypic plasticity by inducing stemness features via Wnt/β-catenin and Hh/GLI1 pathways. This integrative role positions Gal-1 as a key orchestrator of tumor aggressiveness in gastric cancer. **Note:** Gal-1—Galectin-1; MAPK—Mitogen-Activated Protein Kinase; PI3K—Phosphatidylinositol 3-Kinase; AKT—Protein Kinase B; NRP-1—Neuropilin-1; c-JUN—Jun Proto-Oncogene; Wee1—Wee1-like Protein Kinase; TGF-β—Transforming Growth Factor Beta; Hh—Hedgehog; GLI1—Glioma-Associated Oncogene Homolog 1; EMT—Epithelial-to-Mesenchymal Transition; CAFs—Cancer-Associated Fibroblasts; ECM—Extracellular Matrix; NCAPG—Non-SMC Condensin I Complex Subunit G; GRP78—Glucose-Regulated Protein 78; Wnt—Wingless/Integrated (Wg/Int) Signaling Pathway.

A comprehensive overview of Galectin-1-mediated modulation of cancer hallmarks in gastric cancer is provided in Figure 2.

## 4. Galectin-3 in Gastric Cancer: A Multifunctional Driver of Tumor Progression

Galectin-3 (Gal-3) is a unique chimera-type galectin involved in diverse cellular processes, including adhesion, apoptosis resistance, immune modulation, and metastatic spread. Its dynamic localization—cytoplasmic, nuclear, and extracellular—enables Gal-3 to influence signaling both within tumor cells and across the tumor microenvironment. In gastric cancer, Gal-3 expression is frequently upregulated and associated with enhanced malignancy, therapy resistance, and peritoneal dissemination. The following sections explore the structural features, biological functions, and pathological relevance of Gal-3 in the context of gastric tumorigenesis.

### 4.1. Galectin-3: Structure, Localization, and Biological Roles

Galectin-3 is the only chimeric-type member of the galectin family, characterized by a single CRD connected to an N-terminal domain rich in proline and glycine [101]. This unique structure allows Gal-3 to function as a monomer or to form oligomers upon binding to multivalent glycoconjugates, enabling it to crosslink ligands and modulate cellular signaling [102].

Galectin-3 is synthesized on cytoplasmic ribosomes and is primarily localized in the cytoplasm but can translocate to the nucleus and be secreted extracellularly through non-classical pathways [103]. It is broadly expressed in immune cells such as macrophages, neutrophils, mast cells, and Langerhans cells, where it participates in inflammatory and antimicrobial responses [104].

Galectin-3 recognizes complex glycans, especially poly-N-acetyllactosamine structures, and binds to a wide range of extracellular ligands including laminin, fibronectin, tenascin, and integrins (e.g., α1β1 and αMβ2) [105]. Through these interactions, Gal-3 modulates adhesion, migration, and cell–matrix communication. Intracellularly, Gal-3 engages in protein–protein interactions with cytokeratins, B-cell lymphoma 2 (Bcl-2), gem-associated protein 4 (Gemin4), and ALG-2-interacting protein X/apoptosis-linked gene 2-interacting protein 1 (Alix/AIP-1) [105]. In mitochondria, its NWGR motif—shared with Bcl-2 family proteins—confers antiapoptotic activity [106]. In the nucleus, Gal-3 participates in spliceosome regulation via its association with the survival of the motor neuron (SMN)–Gemin complex and Alix/AIP-1, suggesting a role in RNA processing and cell death regulation [105].

Functionally, Gal-3 influences numerous physiological and pathological processes including cell proliferation, differentiation, apoptosis inhibition, pre-mRNA splicing, inflammation, angiogenesis, and host defense [101]. It enhances phagocyte survival, neutrophil extravasation, and fibrotic remodeling, and has been implicated in resistance to apoptosis and promotion of tumor progression. Consequently, Gal-3 has emerged as a potential diagnostic and prognostic biomarker in cardiovascular, renal, and oncologic diseases [107].

### 4.2. Galectin-3 Expression in Gastric Mucosa

In the gastrointestinal tract, Gal-3 is predominantly expressed by epithelial cells, with selective and robust expression in the surface epithelial cells of the gastric mucosa [108]. Immunohistochemical analyses have consistently shown that Gal-3 localization in the stomach is largely restricted to the superficial epithelial layer [109]. Additionally, immunoblot assays have confirmed the presence of Gal-3 not only within gastric tissue but also in the surface mucus layer, indicating active secretion into the luminal interface [109]. These findings suggest that Gal-3 plays a physiological role in maintaining epithelial integrity and contributing to mucosal barrier function in the stomach.

### 4.3. Galectin-3 Expression and Prognostic Significance in Gastric Cancer

In normal gastric mucosa, Gal-3 is predominantly localized in the cytoplasm of surface epithelial cells, with faint or absent nuclear staining. In contrast, GC tissues frequently exhibit both cytoplasmic and nuclear Gal-3 expression, with nuclear localization often associated with actively proliferating tumor cells [110]. Multiple studies have confirmed significantly higher Gal-3 expression in GC tissues compared to adjacent non-malignant mucosa, with increased positivity observed especially in papillary and poorly differentiated adenocarcinomas [110,111,112,113,114,115,116]. Some findings also suggest stronger expression in metastatic lymph nodes relative to primary tumor sites, highlighting Gal-3’s possible role in promoting metastasis [110,117].

Despite substantial variability among studies, Gal-3 expression has been correlated with adverse pathological features, including advanced tumor stage, lymph node metastasis, deeper invasion, and vascular infiltration [114]. However, data on its prognostic value remain inconclusive. While some studies identified no significant association between Gal-3 expression and survival, others reported that decreased Gal-3 expression was independently linked to poor prognosis, particularly in poorly differentiated tumors [111,113,114,118,119]. Meta-analyses have reinforced this, showing that low Gal-3 expression correlates with lymphatic invasion, deeper infiltration, poorer differentiation, and reduced overall survival [120].

Serum Gal-3 levels, however, have limited clinical utility. While some studies noted elevated serum concentrations in GC patients compared to healthy controls or those with benign gastric disease, no consistent association was found between serum Gal-3 levels and tumor characteristics, treatment response, or overall survival [121,122]. Thus, Gal-3 does not appear to be a reliable serological marker for diagnosis or prognosis in gastric cancer.

### 4.4. Galectin-3-Mediated Signaling and Functional Impact in Gastric Cancer Cells

Galectin-3 plays a central role in promoting GC cell proliferation, migration, invasion, and therapeutic resistance through modulation of multiple intracellular signaling pathways [116]. In GC cell lines, Gal-3 enhances motility by upregulating *Fascin-1*, an actin-bundling protein involved in cytoskeletal dynamics [123]. This regulation occurs via Gal-3 interaction with the beta-catenin (β-catenin)/T-cell factor 4 (TCF-4) complex and stabilization of β-catenin through protein kinase B (AKT)-mediated phosphorylation of glycogen synthase kinase-3 beta (GSK-3β) [123]. Fascin-1 expression is further driven by transcription factors such as NF-κB, cAMP response element-binding protein (CREB), and TCF-4, all modulated by Gal-3 activity [123].

Additionally, Gal-3 promotes proliferation and metastasis through the Ras-like protein A (RalA)/Ral-binding protein 1 (RalBP1) complex, enhancing cellular MYC (c-MYC) and Yes-associated protein 1 (YAP1) signaling [116]. Silencing Gal-3 impairs cell migration and alters cell morphology, indicating its essential role in maintaining the invasive phenotype [123].

Galectin-3 also induces immune escape mechanisms; it facilitates resistance to interferon-gamma (IFN-γ) by activating the AKT/GSK-3β/Src homology region 2-containing protein tyrosine phosphatase 2 (SHP2) cascade, leading to suppressed IFN-γ-induced apoptosis [124]. This resistance is reversed upon Gal-3 silencing [124].

Genetic variants in *LGALS3*, such as rs4644 and rs4652, have been linked to altered Gal-3 expression and function [125]. The rs4652 variant, located in the carbohydrate recognition domain, may enhance Gal-3’s interaction with fibroblast receptors, promoting angiogenesis via TGF-β signaling [125]. These single nucleotide polymorphisms (SNPs) have been associated with increased GC risk, tumor progression, and chemoresistance.

Galectin-3 also modulates pro-metastatic mediators such as matrix metalloproteinase-1 (MMP-1) and protease-activated receptor-1 (PAR-1) [126]. It directly interacts with AP-1 components—jun proto-oncogene (c-Jun) and Fos-related antigen 1 (Fra-1)—which activate PAR-1 expression, and subsequently increase MMP-1 production [126]. This dual role in degrading the ECM and promoting receptor signaling enhances GC invasiveness.

Furthermore, Gal-3 upregulates the Hyaluronan-Mediated Motility Receptor (HMMR/CD168), a key regulator of cytoskeletal dynamics and mitotic spindle function [127]. Gal-3 binds to the transcription factor CCAAT/enhancer-binding protein beta (C/EBPβ) and promotes hyaluronan-mediated motility receptor (HMMR) expression, thereby increasing GC cell motility and proliferation [126]. HMMR has also been implicated in chemotherapy resistance and poor outcomes in various cancers, including GC [128].

### 4.5. Galectin-3 in Gastric Cancer Metastasis and Peritoneal Dissemination

Galectin-3 plays a central role in facilitating GC metastasis by disrupting cell–cell and cell–matrix adhesion, promoting cytoskeletal remodeling, and enhancing cell motility [123]. Gal-3 binds integrins such as α1β1, weakening intercellular cohesion and aiding in tumor cell detachment and dissemination [123]. Silencing of Gal-3 results in downregulation of key motility-related proteins, including *MMP-1*, *MMP-3*, *Fascin-1*, and *HMMR*, while increasing inhibitors of matrix degradation, suggesting a pivotal role in extracellular matrix remodeling and invasion [123,127].

Mechanistically, Gal-3 regulates *Fascin-1* expression via the Wnt/β-catenin/TCF-4 axis, enhancing filopodia formation and migratory potential [123]. Galectin-3 also promotes neogenin-1 (NEO1) expression through Rho-associated coiled-coil-containing protein kinase 1 (ROCK1) phosphorylation and supports metastatic behavior via deregulation of actin-related proteins such as annexin A2 (ANXA2), which modulates Rho-dependent cytoskeletal dynamics [123]. These pathways collectively drive aggressive phenotypes in GC cells.

In the context of peritoneal carcinomatosis (PC)—the most common and lethal form of metastasis in gastric adenocarcinoma—Gal-3 plays a particularly important role through its presence in cancer-derived exosomes [24,129]. Exosomal Gal-3 is highly enriched in malignant ascites and acts as a key mediator of peritoneal niche formation [24]. It activates CAFs via the integrin alpha-1 beta-1 (α1β1)/focal adhesion kinase (FAK)/protein kinase B (AKT)/mechanistic target of rapamycin (mTOR)/C-X-C motif chemokine ligand 12 (CXCL12) pathway, promoting immunosuppressive microenvironments through the CXCL12/C-X-C motif chemokine receptor 4 (CXCR4) axis [24]. This facilitates tumor implantation and resistance to immune surveillance.

Targeting exosomal Gal-3, either alone or in combination with CXCL12/CXCR4 blockade, has shown significant potential in experimental models—reducing peritoneal metastases and enhancing responses to anti-PD-1 immunotherapy [24]. Moreover, Gal-3 synergizes with CD47 in peritoneal metastases of diffuse-type GC, contributing to immune evasion [130]. While CD47 inhibits macrophage phagocytosis via signal regulatory protein alpha (SIRPα), Gal-3 reinforces immune suppression by inducing macrophage M2 polarization, promoting CD8+ T-cell apoptosis, and impairing T-cell receptor clustering [130].

Altogether, Gal-3 serves as a master regulator of metastatic progression in GC, especially within the peritoneal cavity. Through its extracellular and exosomal functions, Gal-3 not only facilitates stromal remodeling and immune escape but also presents a promising dual target for therapies aimed at interrupting tumor spread and restoring antitumor immunity.

### 4.6. Galectin-3 and Cellular Senescence in Gastric Cancer

Cellular senescence, characterized by irreversible cell cycle arrest, represents a barrier to uncontrolled proliferation and plays a dual role in tumor suppression and progression [131]. In gastric cancer, Gal-3 has emerged as a critical regulator of both replicative and premature senescence [132,133]. Galectin-3 ablation has been shown to downregulate human telomerase reverse transcriptase (hTERT), a pivotal enzyme required for telomere maintenance and cancer cell immortality [132]. Experimental data indicate a bidirectional regulatory relationship between Gal-3 and hTERT: knockdown of one leads to downregulation of the other, and Gal-3 deficiency-induced senescence can be reversed through forced hTERT overexpression [132]. Mechanistically, immunoprecipitation assays have revealed that Gal-3 directly binds to the N-terminal region of hTERT, thereby modulating its stability and activity [132].

In addition to its role in replicative senescence, Gal-3 is implicated in the prevention of premature senescence [133]. Depletion of Gal-3 results in decreased expression of S-phase kinase-associated protein 2 (Skp2) and increased accumulation of the cyclin-dependent kinase inhibitor p27^KIP1^, leading to G1 cell cycle arrest. This process is mediated through reduced formation and activity of the *cyclin D1/CDK4* complex, resulting in hypophosphorylation of the retinoblastoma (Rb) protein—a hallmark of senescence induction [133]. Conversely, Gal-3 enhances cyclin D1/CDK4 activity and promotes Rb hyperphosphorylation, thereby supporting cell cycle progression and circumventing senescence.

Furthermore, Gal-3 promotes Skp2 expression, which in turn facilitates post-translational degradation of p27^KIP1^, without affecting its mRNA transcription [133]. Skp2, a known oncogene in gastric cancer, thus acts in concert with Gal-3 to repress senescence and promote tumorigenesis [134]. Collectively, these findings highlight Gal-3 as a central orchestrator of senescence escape in gastric cancer cells, enabling sustained proliferation through hTERT stabilization, Rb pathway modulation, and regulation of key cell cycle mediators such as Skp2 and p27^KIP1^.

### 4.7. Galectin-3 and Chemoresistance in Gastric Cancer

Chemotherapeutic resistance remains a major barrier to successful treatment of gastric cancer, and Gal-3 has emerged as a key contributor to this phenomenon [135]. Overexpression of Gal-3 is associated with reduced sensitivity to chemotherapeutic agents, largely due to its ability to regulate genes involved in apoptosis and cell survival [136]. Gal-3 exerts these effects by translocating into the nucleus and directly interacting with transcription factors that modulate the expression of antiapoptotic proteins such as baculoviral inhibitor of apoptosis repeat-containing 5 (survivin), X-linked inhibitor of apoptosis protein (XIAP), and XIAP-associated factor 1 (XAF-1), thereby promoting cell survival under cytotoxic stress [136].

**Figure 3:**
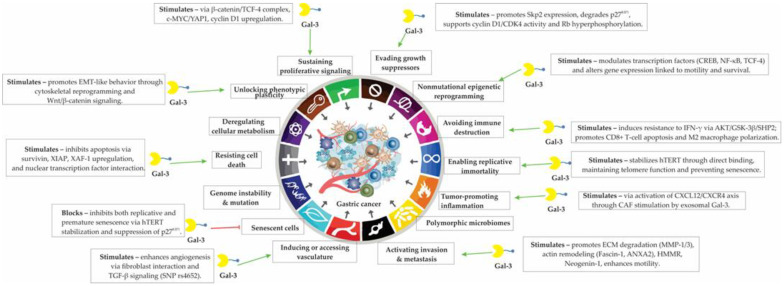
Galectin-3 as a multifunctional promoter of tumor progression pathways in gastric cancer. This figure illustrates the multifaceted role of Gal-3 in promoting various cancer-associated biological programs in gastric adenocarcinoma. Gal-3 enhances proliferative signaling via β-catenin/TCF-4 and c-MYC/YAP1 pathways, supports cell cycle progression through Skp2-mediated degradation of p27^KIP1^ and cyclin D1/CDK4 activation, and enables resistance to apoptosis by upregulating survivin, XIAP, and XAF-1. It facilitates immune evasion via CD8+ T-cell suppression and M2 macrophage polarization, drives EMT and metastatic behavior by modulating Fascin-1, ANXA2, HMMR, and Neogenin-1, and promotes angiogenesis through TGF-β signaling. Gal-3 also sustains replicative immortality by stabilizing hTERT and modulates senescence and chemoresistance via the Rb/p27^KIP1^ axis and Wnt/β-catenin signaling. Through these mechanisms, Gal-3 acts as a central regulator of tumor progression, therapeutic resistance, and metastatic dissemination in gastric cancer. **Note:** Gal-3—Galectin-3; TCF-4—T-cell Factor 4; c-MYC—Myelocytomatosis Viral Oncogene Homolog; YAP1—Yes-Associated Protein 1; Skp2—S-phase Kinase-Associated Protein 2; p27^KIP1—Cyclin-Dependent Kinase Inhibitor 1B; CDK4—Cyclin-Dependent Kinase 4; Rb—Retinoblastoma Protein; XIAP—X-linked Inhibitor of Apoptosis Protein; XAF-1—XIAP-Associated Factor 1; CREB—cAMP Response Element-Binding Protein; NF-κB—Nuclear Factor Kappa-light-chain-enhancer of Activated B cells; AKT—Protein Kinase B; GSK-3β—Glycogen Synthase Kinase 3 Beta; SHP2—Src Homology Region 2-containing Protein Tyrosine Phosphatase-2; hTERT—Human Telomerase Reverse Transcriptase; CXCL12—C-X-C Motif Chemokine Ligand 12; CXCR4—C-X-C Motif Chemokine Receptor 4; ECM—Extracellular Matrix; MMP-1/3—Matrix Metalloproteinases 1 and 3; ANXA2—Annexin A2; HMMR—Hyaluronan-Mediated Motility Receptor; EMT—Epithelial-to-Mesenchymal Transition; TGF-β—Transforming Growth Factor Beta.

In gastric cancer, Gal-3 has been detected in both primary tumors and metastatic lymph nodes, further underscoring its role in disease progression and therapeutic resistance. One of the critical pathways regulated by Gal-3 involves β-catenin/TCF-4, a transcriptional complex that controls the expression of cyclin D1 [136]. Galectin-3 directly binds to this complex, facilitating the upregulation of cyclin D1, and thus, enhancing proliferation and resistance to chemotherapeutic-induced cell cycle arrest. Silencing of Gal-3 leads to cyclin D1 downregulation, increased apoptosis, and improved response to chemotherapeutic agents [136]. The diverse pro-tumorigenic functions of Gal-3 across multiple cellular programs relevant to gastric cancer are summarized in Figure 3.

## 5. Galectin-9 in Gastric Cancer: A Context-Dependent Modulator of Tumor Progression and Immunity

Galectin-9 (Gal-9) is a tandem-repeat lectin with complex and dualistic roles in tumor progression and immune regulation. In gastric cancer, Gal-9 exhibits both antitumorigenic and immunosuppressive activities, depending on expression levels, cellular context, and tumor stage. It modulates proliferation, apoptosis, and epithelial–mesenchymal transition, while also interacting with immune checkpoint molecules—particularly TIM-3—to regulate T-cell exhaustion and immune evasion. Elevated Gal-9 expression correlates with favorable prognosis and reduced metastatic potential, yet its upregulation following chemoradiotherapy may contribute to adaptive immune resistance. These multifaceted functions make Gal-9 a compelling candidate for prognostic evaluation and targeted immunotherapy in gastric cancer.

### 5.1. Galectin-9: A Dual Regulator of Tumor Progression and Immune Surveillance

Galectin-9, a tandem-repeat β-galactoside-binding lectin encoded by *LGALS9*, has emerged as a multifaceted regulator with significant roles in both tumor biology and immune modulation [137]. Originally characterized as a transmembrane urate transporter and eosinophil chemoattractant, Gal-9 is now recognized for its potent immunosuppressive activity and diverse physiological functions, including regulation of cell adhesion, proliferation, differentiation, apoptosis, and intercellular communication [137,138].

In the context of cancer, aberrant expression of Gal-9 has been reported in a variety of solid tumors and is frequently associated with tumor progression, metastasis, and immune evasion [139,140]. Its immunomodulatory effects are primarily mediated through interaction with the checkpoint receptor T-cell immunoglobulin and mucin-domain containing-3 (TIM-3), leading to apoptosis of cluster of differentiation 8-positive (CD8^+^) T cells and suppression of T-helper 1 (Th1) and T-helper 17 (Th17) responses, and expansion of regulatory T cells (Tregs) [141,142]. These mechanisms contribute to an immunosuppressive tumor microenvironment and represent a barrier to effective antitumor immunity. Notably, Gal-9 has been proposed as a novel immune checkpoint molecule, functioning in a manner analogous to the programmed cell death protein 1 (PD-1)/programmed death-ligand 1 (PD-L1) axis, and is currently under investigation as a therapeutic target in immune checkpoint blockade strategies [138].

Galectin-9 exerts concentration-dependent effects on T-cell function: while low concentrations may enhance cytokine secretion by activated T cells, higher concentrations induce apoptosis, particularly of effector CD4^+^ and CD8^+^ subsets. In addition to T-cell regulation, Gal-9 also modulates the behavior of other immune cells. It impairs B-cell signaling, promotes dendritic cell (DC) maturation via upregulation of co-stimulatory molecules—cluster of differentiation 40 (CD40), cluster of differentiation 80 (CD80), and human leukocyte antigen—DR isotype (HLA-DR)—and influences DC cytoskeletal integrity, affecting antigen presentation and phagocytic capacity [138].

Furthermore, Gal-9 has been implicated in processes critical for tumor immune escape, including angiogenesis, autophagy, lysosomal regulation, and maintenance of epithelial barrier function [138]. Importantly, its intracellular roles include regulation of lysosomal stability and cortical actin dynamics, particularly in antigen-presenting cells [143]. Emerging evidence also points to Gal-9 as a potential biomarker for treatment stratification in immunotherapy-resistant tumors, including gastric cancer [138].

### 5.2. Antitumor Activity of Galectin-9 in Gastric Cancer Cell Lines

Galectin-9 has demonstrated notable antitumor effects in gastric cancer cell lines, particularly by suppressing cellular proliferation and inducing apoptosis [144]. In vitro studies using the MKN74 gastric cancer cell line revealed that recombinant human Gal-9 (rh-Gal-9) significantly reduces cell viability by triggering apoptotic pathways [144]. These effects appear to be mediated, at least in part, through the downregulation of phosphorylated insulin-like growth factor 1 receptor (phospho-IGF-1R), suggesting a potential role for Gal-9 in enhancing chemosensitivity and improving clinical outcomes [144].

Moreover, Gal-9 influences multiple intracellular signaling cascades, including receptor tyrosine kinase (RTK) pathways and the expression of angiogenesis-related molecules [144]. Transcriptomic profiling has further shown that Gal-9 alters miRNA expression patterns, implying broader epigenetic and transcriptional regulation [144].

### 5.3. Clinical Significance of Galectin-9 Expression in Gastric Cancer

Galectin-9 expression has been shown to differ significantly between gastric cancer lesions and adjacent normal gastric mucosa, with most tumor cells exhibiting strong cytoplasmic Gal-9 immunoreactivity [145]. Western blot analyses confirmed elevated Gal-9 levels in tumor tissues relative to non-neoplastic epithelium, suggesting its potential involvement in tumor-host interactions [145]. However, conflicting data exist regarding Gal-9 mRNA expression, with some studies reporting its downregulation in gastric cancer specimens, indicating possible post-transcriptional regulatory mechanisms [144].

Immunohistochemical studies have shown that up to 86.2% of gastric cancer patients display Gal-9 positivity within tumor cells [145]. Importantly, higher Gal-9 expression was significantly associated with favorable clinicopathological features, including the absence of lymphovascular invasion, lymph node metastasis, and distant dissemination [144]. Furthermore, patients with high Gal-9 expression exhibited improved overall survival, positioning Gal-9 as a potential prognostic biomarker.

In a meta-analysis comprising 2093 patients from eight independent studies, low Gal-9 expression was consistently linked to poorer prognosis [120]. TNM-based stratification further revealed correlations between Gal-9 expression and tumor stage, pT classification, and lymph node status, though no statistically significant association was observed with distant metastasis [146]. No differences in Gal-9 expression were found based on sex, histological differentiation, or survival time categories [147].

These findings suggest that Gal-9 may exert antitumor activity in vivo and that its loss may facilitate disease progression, highlighting its potential utility as a prognostic indicator and therapeutic target in gastric cancer.

### 5.4. Immunoregulatory Role of the Galectin-9/Tim-3 Axis in Gastric Cancer

The Tim-3/Gal-9 pathway has emerged as a key modulator of Th1-mediated immunity [145]. Tim-3, a cell surface receptor predominantly expressed on Th1 cells, contributes to immune homeostasis and peripheral tolerance [148]. Galectin-9, its high-affinity ligand, binds to Tim-3 and induces apoptosis of Th1 cells, thereby dampening pro-inflammatory immune responses [145].

In gastric cancer, an imbalance in this pathway—characterized by reduced Gal-9 expression and upregulated Tim-3—has been associated with unfavorable clinical outcomes [145]. The loss of Gal-9 weakens its immunosuppressive interaction with Tim-3, potentially resulting in unchecked Tim-3–driven T cell exhaustion and impaired antitumor immunity [145]. These findings suggest that dysregulation of the Gal-9/Tim-3 axis may contribute to immune evasion in gastric cancer and hold promise as a target for immunotherapeutic intervention.

### 5.5. Galectin-9 Upregulation as a Resistance Mechanism to Chemoradiotherapy in Gastric Cancer

Recent findings underscore that chemoradiotherapy induces immunogenic cell death (ICD) in GC cell lines, as evidenced by the translocation of calreticulin (CRT) to the plasma membrane [148]. However, in parallel with this pro-immunogenic signal, GC cells also demonstrate upregulation of immune checkpoint molecules such as PD-L1 and Gal-9e, suggesting an adaptive resistance mechanism [149].

Galectin-9, a β-galactoside-binding lectin, plays a critical immunoregulatory role by engaging the Tim-3 receptor on T cells and dendritic cells [145]. This interaction leads to T cell exhaustion, apoptosis of cytotoxic lymphocytes, and suppression of dendritic cell function, all of which contribute to immune evasion. The increased expression of Gal-9 following chemoradiation—either in soluble or exosomal form—can facilitate reattachment to cancer cells and further dampen antitumor immune responses [148].

This Gal-9 upregulation appears to be stress-inducible and may be linked to endoplasmic reticulum (ER) stress, a response frequently triggered by chemoradiation. In this context, Gal-9 expression may function in tandem with PD-L1 to create a potent immune-suppressive tumor microenvironment, thereby limiting the efficacy of immune-mediated cytotoxicity.

### 5.6. Galectin-9 Suppresses EMT and Metastatic Progression in Gastric Cancer

Emerging evidence identifies Gal-9 as a negative regulator of epithelial–mesenchymal transition in gastric cancer [150]. As a downstream effector of peroxisome proliferator-activated receptor γ (PPARγ), Gal-9 inhibits key steps in tumor progression, including ECM invasion, cellular detachment from the primary tumor, and adherence to the vascular endothelium. Ref. [22] Loss of Gal-9 expression correlates with increased cancer cell invasiveness and migratory capacity, translating clinically into more advanced tumor (T) and nodal (N) stages.

**Figure 4:**
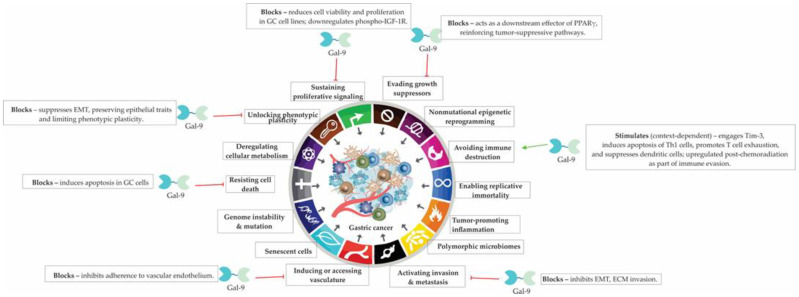
Galectin-9 as a multifunctional modulator of tumor suppression and immune evasion in gastric cancer. This figure depicts the dual, context-dependent role of Galectin-9 (Gal-9) in gastric cancer pathogenesis. Gal-9 suppresses key hallmarks of cancer by inhibiting proliferative signaling, inducing apoptosis, and preserving epithelial traits to prevent EMT and metastasis. It also blocks vascular adhesion and ECM invasion, reinforcing its tumor-suppressive profile. However, Gal-9 engages Tim-3 on Th1 and dendritic cells, promoting immune suppression, T cell exhaustion, and resistance to therapy. Its expression is often elevated post-chemoradiation as part of an adaptive immune evasion mechanism. Through these diverse mechanisms, Gal-9 serves as both a prognostic biomarker and a potential therapeutic target depending on tumor stage, localization, and treatment context. **Note**: Gal-9—Galectin-9; EMT—Epithelial-to-Mesenchymal Transition; ECM—Extracellular Matrix; IGF-1R—Insulin-like Growth Factor 1 Receptor; PPARγ—Peroxisome Proliferator-Activated Receptor Gamma; Tim-3—T-cell Immunoglobulin and Mucin-domain Containing-3; Th1—T helper type 1.

Patients with Gal-9-positive tumors exhibit significantly improved survival outcomes compared to Gal-9-negative cases, which may reflect reduced metastatic potential associated with sustained Gal-9 expression. Mechanistically, Gal-9 impairs EMT by preserving epithelial characteristics and suppressing adhesion to ECM components, thereby disrupting the critical steps required for metastatic dissemination and organ colonization (Figure 4).

## 6. Galectin-2 in Gastric Physiology and Cancer: A Context-Dependent Regulator

Galectin-2 (Gal-2), a lesser-studied member of the galectin family, is predominantly expressed in the gastrointestinal tract, particularly in gastric epithelial cells. Structurally similar to Gal-1, yet functionally distinct, Gal-2 plays key roles in mucosal protection, immune regulation, and epithelial integrity. In the gastric environment, it contributes to barrier function through interactions with mucins, while its downregulation in gastric cancer has been linked to disease progression and poorer prognosis. These dual roles underscore the context-dependent nature of Gal-2 in health and malignancy.

### 6.1. Structural and Functional Characteristics of Galectin-2

Galectin-2 is a member of the galectin family that shares approximately 43% amino acid sequence identity with galectin-1 [151,152]. Structurally, Gal-2 is a 14 kDa protein containing a single CRD located at its C-terminal [153]. Although it exists predominantly as a monomer, Gal-2 can form non-covalent homodimers in solution, allowing it to crosslink glycosylated receptors on the cell surface and modulate a variety of cellular functions [153].

Synthesized in the cytoplasm, Gal-2 can translocate to the nucleus, cell membrane, or extracellular space [154]. Similar to other galectins, Gal-2 lacks a classical secretion signal peptide and is believed to be secreted via non-conventional, possibly endosome-mediated pathways. Nuclear localization of Gal-2 may occur through passive diffusion or via interaction with nuclear transport proteins, as described for galectin-3 [153].

Gal-2 exhibits strong glycan-binding specificity, particularly for complex galactose-terminated oligosaccharides such as N-acetyllactosamine (LacNAc), to which it binds with much higher affinity than to galactose monosaccharides or simple disaccharides [155]. Known glycoprotein ligands of Gal-2 include β1 integrin on T cells, mucin 1 (MUC1) in epithelial cancer cells, mucin 5AC (MUC5AC) in gastric mucosa, ganglioside monosialotetrahexosylganglioside 1 (GM1) in neuroblastoma cells, and other molecules such as lymphotoxin-α and β-tubulin, particularly in inflammatory contexts like atherosclerotic plaques [153].

Functionally, Gal-2 participates in the regulation of epithelial barrier integrity, immune responses, inflammation, and apoptosis [153]. Its immunomodulatory role has been demonstrated in several models, including suppression of contact hypersensitivity, promotion of apoptosis in activated T lymphocytes, and modulation of inflammatory pathways in colitis. It also plays a beneficial role in mucosal healing processes [151,152,156].

Altered Gal-2 expression has been observed in various pathological conditions, including inflammatory bowel disease, pregnancy-related disorders, and several cancers [153]. These observations point to a context-dependent role for Gal-2, where it may act either as a mediator of homeostasis or a contributor to disease progression, depending on the tissue and disease microenvironment.

### 6.2. Galectin-2 Expression and Function in Gastric Epithelium

Galectin-2 is predominantly expressed in the gastrointestinal tract, with particularly high levels found in epithelial cells of the stomach, including mucous neck cells and surface mucous cells [108,153,157]. In murine models, Gal-2 secreted by gastric epithelial cells binds to the mucin glycoprotein MUC5AC, facilitating the crosslinking of mucin molecules within the mucus layer [158]. This interaction enhances the structural integrity of the gastric mucus barrier, contributing to epithelial protection by reinforcing the physical defense against luminal insults and microbial invasion.

### 6.3. Galectin-2 Downregulation in Gastric Cancer and Its Association with Prognosis

**Figure 5:**
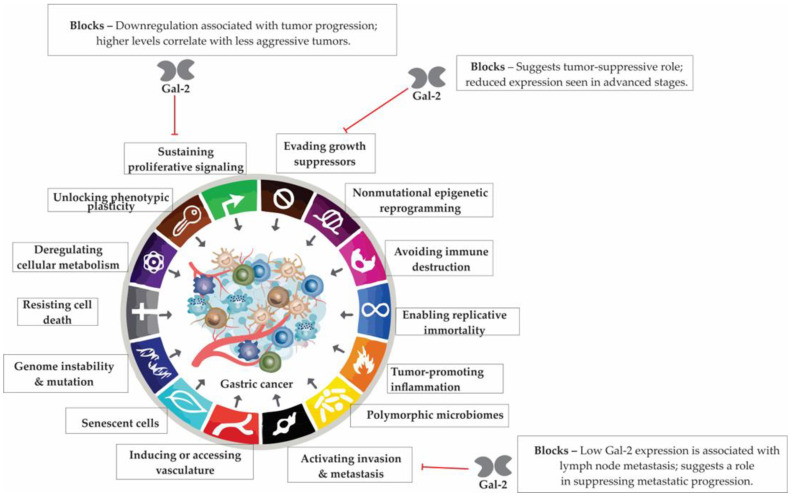
Galectin-2 as a potential tumor suppressor across gastric cancer hallmarks. This schematic illustrates the inhibitory role of Gal-2 in gastric cancer progression. Reduced expression of Gal-2 has been associated with advanced disease stages, lymph node metastasis, and more aggressive tumor phenotypes. Gal-2 is shown to negatively regulate several hallmarks of cancer, including proliferative signaling, evasion of growth suppressors, and metastatic dissemination.

Helicobacter pylori infection is a well-established environmental risk factor for non-cardia gastric cancer [159]. Experimental models have demonstrated that Gal-2 expression is significantly reduced in H. pylori-induced gastric cancer lesions compared to normal gastric tissue, suggesting a potential tumor-suppressive role [160]. In human GC, decreased Gal-2 expression has been associated with disease progression, particularly in cases with lymph node metastasis (LNM)—a key prognostic determinant in GC. Notably, Gal-2 levels were found to be up to 12-fold higher in tumor tissues without LNM compared to those with advanced disease and nodal involvement [161]. These findings imply that preserved or elevated Gal-2 expression may be indicative of less aggressive tumor behavior and could be linked to more favorable clinical outcomes in gastric cancer patients (Figure 5).

## 7. Galectin-4 in Gastric Cancer: Linking Epithelial Biology to Tumor Progression

Galectin-4 (Gal-4), a tandem-repeat lectin primarily expressed in gastrointestinal epithelia, plays a dual role in maintaining mucosal homeostasis and promoting malignant transformation. Through its two carbohydrate-recognition domains, Gal-4 modulates membrane organization, glycan-dependent adhesion, and intracellular signaling. While physiologically involved in barrier integrity and immune defense, aberrant Gal-4 expression in gastric cancer has been linked to enhanced proliferation, receptor activation, and peritoneal dissemination, underscoring its emerging relevance as a biomarker and potential therapeutic target.

### 7.1. Galectin-4: Structure, Localization, and Functional Roles in Gastrointestinal Physiology and Pathology

Galectin-4 is a member of the tandem-repeat galectin subfamily, characterized by two CRDs located within a single polypeptide chain [162]. These CRDs share approximately 40% sequence homology but exhibit distinct ligand specificities, allowing Gal-4 to function as a molecular crosslinker with the capacity to mediate complex cellular interactions [163]. Galectin-4 is predominantly expressed by epithelial cells of the intestinal tract and was initially identified as a 17 kDa protein in extracts from rat small intestine [164].

Functionally, Gal-4 is distributed intracellularly, on the cell surface, and in the extracellular environment, despite lacking a classical signal peptide for ER-mediated secretion. Its presence on the cell membrane and in circulation is attributed to secretion via non-classical pathways [163]. Intracellularly, Gal-4 modulates cellular processes such as proliferation, apoptosis, and differentiation, whereas extracellular Gal-4 is involved in intercellular adhesion by binding β-galactoside-containing ligands, including glycoproteins, blood group antigens, MUC1-like mucins, and glycosphingolipids [163,165,166].

Beyond its adhesive properties, Gal-4 enhances lipid raft stabilization, which is crucial for signal transduction and membrane organization. Additionally, Gal-4 has been reported to exhibit bactericidal activity against microbes expressing blood group antigens, suggesting a role in innate immune defense. Recent data also highlight its importance in mucosal repair; Gal-4 promotes intestinal wound healing and may participate in the resolution of epithelial damage [163].

Ongoing research has increasingly focused on the contribution of Gal-4 to gastrointestinal homeostasis, inflammation, and malignancy.

### 7.2. Galectin-4 in Gastric Cancer Progression and Peritoneal Dissemination

In gastric cancer, elevated expression of Gal-4 has been identified as an independent predictor of metastasis and poor clinical outcomes, functioning as a tumor promoter particularly in aggressive and poorly differentiated tumors [167]. Gal-4 is a β-galactoside-binding lectin that plays a multifaceted role in tumor biology, including modulation of glycosylation, membrane receptor dynamics, and intracellular signaling [102].

Gal-4 is a key regulator of glycosphingolipid (GSL) composition in cancer cells, and its expression has been associated with significant alterations in the glycan profile of malignant cells with a high metastatic potential [168]. Notably, in gastric cancer cell lines such as NUGC4, elevated Gal-4 levels facilitate the formation of glycan-mediated lattices by binding to sulfated GSLs on the cell membrane. This interaction is thought to affect endocytic trafficking and stabilize receptor clusters, thereby promoting abnormal proliferative signaling and enhancing tumor cell dissemination [168].

**Figure 6:**
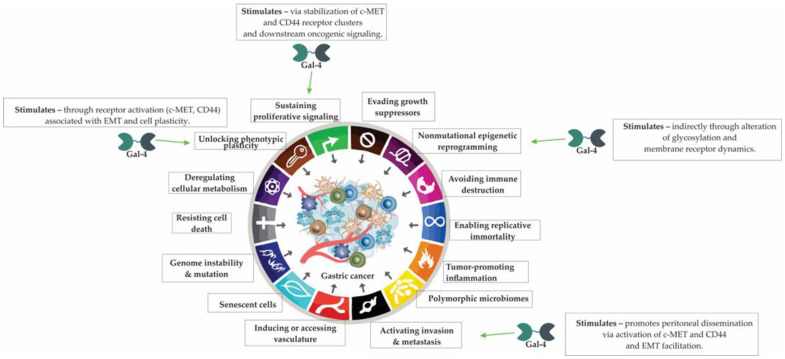
Galectin-4 drives oncogenic signaling and metastatic behavior in gastric cancer. This figure depicts the pro-tumorigenic roles of Gal-4 in gastric cancer, highlighting its stimulatory effects on key biological processes that underlie tumor progression and dissemination. Gal-4 enhances proliferative signaling through stabilization and activation of c-MET and CD44 receptor clusters, modulates non-mutational epigenetic programs by altering glycosylation patterns and membrane receptor dynamics, and promotes cell plasticity and EMT through receptor-driven signaling. Additionally, Gal-4 facilitates metastatic spread, particularly peritoneal dissemination, by supporting invasive behavior and sustained oncogenic signaling. Through these mechanisms, Gal-4 emerges as a significant contributor to aggressive tumor phenotypes and a potential therapeutic target in gastric cancer. **Note:** Gal-4—Galectin-4; c-MET—Mesenchymal–Epithelial Transition Factor; CD44—Cluster of Differentiation 44; EMT—Epithelial-to-Mesenchymal Transition.

Galectin-4 further contributes to peritoneal metastasis through interactions with specific membrane proteins such as mesenchymal–epithelial transition factor (c-MET) and CD44, both of which are known to be involved in gastric cancer progression and epithelial-to-mesenchymal transition [169]. Galectin-4 binding to these receptors, mediated via its carbohydrate recognition domains, enhances receptor activation and downstream oncogenic signaling [169]. Experimental silencing or knockout of Gal-4 in gastric cancer models has been shown to reduce the levels of activated c-MET and CD44, leading to attenuated cell proliferation and decreased peritoneal dissemination (Figure 6) [169].

## 8. Galectin-7 in Gastric Cancer: A Methylation-Silenced Tumor Suppressor with Contextual Duality

Galectin-7 (Gal-7) exhibits distinct, context-dependent roles in cancer biology, functioning either as a tumor suppressor or promoter depending on the tissue type and molecular context. In gastric cancer, emerging evidence points toward a predominantly suppressive function, with marked downregulation of Gal-7 linked to promoter hypermethylation and reduced tumor cell apoptosis. While better known for its involvement in epithelial homeostasis and stress responses, Gal-7’s precise role in gastric tumorigenesis is only beginning to be defined—offering a novel target for further investigation in both prognostic and therapeutic settings.

### 8.1. Galectin-7: Context-Dependent Roles in Cancer Biology

Galectin-7, a member of the galectin family with a single CRD, functions as a homodimer and displays relatively lower affinity for β-galactoside-containing oligosaccharides compared to Gal-1 and -3 [170]. Unlike its better-characterized counterparts, the biological functions of Gal-7 remain only partially understood [171]. It is present in both the cytoplasm and nucleus, as well as at intercellular junctions, suggesting that its function may vary with subcellular localization [171]. Galectin-7 has been implicated in epithelial homeostasis, playing roles in epithelial differentiation, migration, and wound healing [172]. Additionally, it contributes to the regulation of apoptosis, involving mechanisms such as JNK pathway activation, cytochrome c release, and mitochondrial Bcl-2 modulation [173,174,175].

Intriguingly, Gal-7 demonstrates a dual role in cancer biology [171]. In some malignancies, it acts as a tumor suppressor: it has been identified as a p53-induced gene (PIG1), suppresses neuroblastoma proliferation, and enhances cancer cell sensitivity to apoptosis [176]. Conversely, pro-tumorigenic roles have also been reported. Overexpression of Gal-7 has been associated with enhanced metastatic potential in breast and lymphoma models, driven in part by upregulation of MMP-9 and other invasion-related genes [177]. These opposing effects underscore its tissue-specific functionality and complex involvement in tumorigenesis.

Despite the growing body of evidence on Gal-7 in various cancers, its role in gastric cancer remains largely unexplored. This represents a significant knowledge gap and a potential avenue for future investigation, particularly given the importance of other galectins (such as Gal-1, Gal-3, and Gal-4) in gastric tumor progression and metastasis.

### 8.2. Galectin-7: A Methylation-Regulated Tumor Suppressor in Gastric Cancer

Galectin-7 has emerged as a potential tumor suppressor in gastric cancer [178]. Its expression is significantly downregulated in malignant gastric tissues compared to adjacent normal mucosa [178]. Functional studies have demonstrated that Gal-7 overexpression inhibits proliferation, migration, and invasion of AGS gastric cancer cells, whereas its knockdown in KATO III cells enhances these malignant phenotypes [178]. These findings strongly suggest that galectin-7 acts as a suppressor of gastric cancer progression.

However, the functional role of Gal-7 in cancer remains controversial. Initially characterized as a p53-induced, pro-apoptotic protein, Gal-7 has also been implicated in tumor-promoting roles in other malignancies, such as breast cancer and lymphoma, where it contributes to metastasis through the upregulation of MMP-9 [173,179]. These context-dependent effects underscore the complexity of Gal-7’s function and its potential tissue-specific regulatory mechanisms.

**Figure 7:**
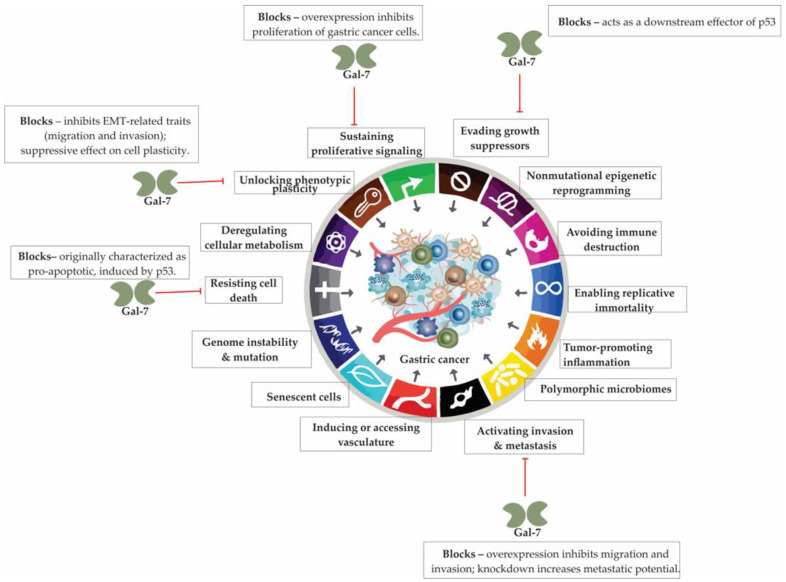
Galectin-7 as a methylation-silenced tumor suppressor modulating cancer hallmarks in gastric cancer. This figure summarizes the inhibitory effects of Gal-7 on several hallmarks of cancer in gastric adenocarcinoma. Gal-7 blocks sustained proliferative signaling, suppresses epithelial–mesenchymal transition and cell plasticity, and inhibits invasion and metastasis. Originally identified as a pro-apoptotic, p53-induced protein, Gal-7 also promotes apoptosis and acts downstream of p53 to reinforce tumor suppression. Its overexpression impairs cancer cell migration and proliferation, whereas its loss—commonly through promoter hypermethylation—removes these inhibitory effects, enabling disease progression. Through its influence on multiple cancer-driving processes, Gal-7 emerges as a clinically relevant tumor suppressor with diagnostic and prognostic potential in gastric cancer. **Note:** Gal-7—Galectin-7; EMT—Epithelial-to-Mesenchymal Transition; p53—Tumor Protein p53.

In gastric cancer, Gal-7 expression is suppressed by promoter hypermethylation [178]. Epigenetic silencing of Gal-7 may reflect broader patterns of tumor suppressor gene inactivation common in gastric tumorigenesis. Notably, the hypermethylated region encompasses a predicted p53 binding site, supporting previous findings that Gal-7 is a downstream effector of p53 signaling [178]. This epigenetic regulation highlights Gal-7 as not only a functional tumor suppressor but also a promising candidate biomarker for early detection and prognostication in gastric cancer (Figure 7).

## 9. Galectin-8 in Gastric Cancer: A Dual-Role Modulator of Tumor Behavior

Galectin-8 (Gal-8) is a multifunctional lectin involved in cell adhesion, immune regulation, and intracellular signaling, exhibiting both tumor-promoting and tumor-suppressive properties depending on context. While Gal-8 overexpression is associated with progression in various cancers, its role in gastric cancer appears more nuanced. Elevated Gal-8 expression in non-metastatic gastric tumors correlates with favorable clinical features and improved survival, suggesting a suppressive function in early disease stages. These context-dependent effects position Gal-8 as both a mechanistic regulator and a promising prognostic biomarker in gastric cancer.

### 9.1. Galectin-8: A Multifunctional Regulator in Cancer Progression

Galectin-8, encoded by the *LGALS8* gene, is a tandem-repeat-type galectin containing two CRDs connected by a variable linker peptide [180]. Its unique glycan-binding specificity enables differential interaction with branched N-glycans and sialylated β-galactosides, supporting roles in cell adhesion, migration, angiogenesis, and immune modulation. Gal-8 is secreted via a non-classical pathway and is detectable in body fluids, including serum and synovial fluid [180].

Functionally, Gal-8 interacts with integrins, CD44, and other adhesion molecules, promoting tumor cell adhesion and dissemination [181]. Intracellularly, it regulates autophagy, inhibits mechanistic target of rapamycin (mTOR) signaling during membrane stress, and suppresses K-Ras activation, linking it to homeostatic repair processes. Gal-8 amplification and overexpression have been observed in several malignancies (breast, lung, prostate), often correlating with poor prognosis. Additionally, Gal-8 modulates cytokine and chemokine networks by influencing NF-κB signaling, thereby contributing to a pro-tumorigenic inflammatory microenvironment. Through these diverse mechanisms, Gal-8 supports tumor progression and metastasis [181].

### 9.2. Prognostic Significance of Galectin-8 in Gastric Cancer

Galectin-8 has emerged as a potential prognostic biomarker in patients with non-metastatic gastric cancer following surgical treatment [180]. Studies have shown that higher intratumoral expression of galectin-8 is significantly correlated with smaller tumor size, reduced recurrence rates, and improved overall survival. This suggests that galectin-8 may play a tumor-suppressive role in the early stages of gastric cancer by modulating cellular adhesion, immune responses, and intracellular signaling pathways [180].

**Figure 8:**
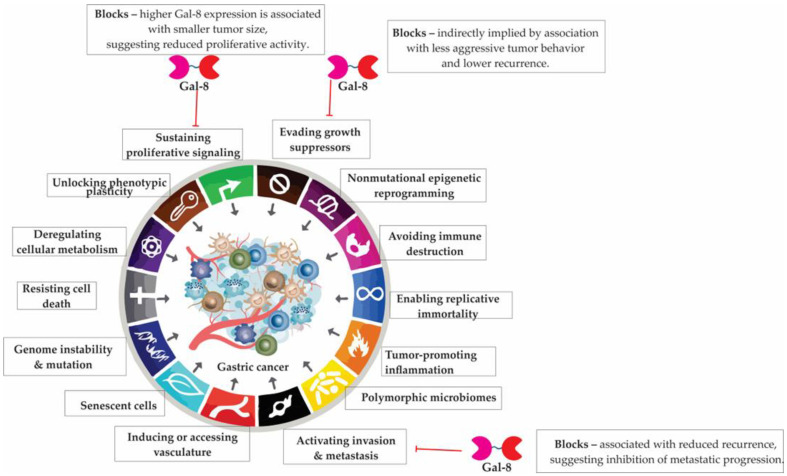
Galectin-8 expression correlates with reduced malignancy and inhibition of key cancer traits in gastric cancer. This figure illustrates the inverse association between Gal-8 expression and several malignant features in gastric cancer. Higher Gal-8 levels are linked to reduced proliferative signaling, suggesting decreased tumor growth potential. Additionally, Gal-8 expression is associated with inhibition of metastatic progression, reflected by lower recurrence rates and less aggressive tumor behavior. These observations support the tumor-suppressive role of Gal-8, particularly in early-stage, non-metastatic gastric cancer, and highlight its clinical relevance as a prognostic biomarker for patient stratification following surgical resection.

Importantly, multivariate analyses have identified high galectin-8 expression as an independent favorable prognostic factor, meaning its predictive value remains significant even when accounting for other clinicopathological variables [180]. Thus, galectin-8 expression levels may serve not only as a biological marker of less aggressive disease but also as a tool to stratify patients in terms of their risk of recurrence and long-term outcome after surgery (Figure 8).

To facilitate comparison and integration of the diverse roles of individual galectins in gastric cancer, we provide a comprehensive summary in Table 1. This table highlights the expression patterns, molecular functions, impact on tumor behavior, clinical correlations, and translational potential of each galectin discussed in this review.

## 10. Conclusions

Accumulating data place galectins at the center of a multifaceted regulatory network that underwrites virtually every hallmark of gastric malignancy, redefining them from ancillary glycan readers to pivotal architects of tumorbehavior. Their dual cellular localization and capacity to cross-link glycoproteins and influence both cancer cells and stromal elements endow galectins with a breadth of influence unmatched by many traditional oncogenic drivers. Recognizing galectins as actionable nodes in gastric cancer biology opens a timely therapeutic window: a strategic blockade of their glycan interactions may enhance cytotoxic efficacy, restore immune competence, and blunt metastatic dissemination. Concerted translational efforts, coupled with refined biomarker development for patient selection, are now required to transform these promising molecular insights into tangible survival benefits for patients afflicted by this challenging disease.

## Figures and Tables

**Table 1:** Summary of galectin family members in gastric cancer: expression profiles, functional roles, clinical implications, and therapeutic potential.

Galectin	Expression in Gastric Cancer	Molecular Functions	Impact on Tumor Progression	Clinical Significance	Role in Treatment Resistance	Therapeutic Potential	Biomarker Potential
**Galectin-1**	Strongly upregulated in stroma and CAFs; moderate in epithelium	Modulates ECM remodeling, angiogenesis, immune evasion, EMT	Promotes proliferation, invasion, metastasis, and immunosuppression	Correlates with advanced stage, poor prognosis	Confers cisplatin resistance via PI3K/AKT and MAPK signaling	Target for antiangiogenic and immune-restorative therapies	Prognostic biomarker of aggressive disease
**Galectin-2**	Downregulated in tumors with lymph node metastasis	Supports epithelial barrier and mucosal homeostasis	Loss promotes mucosal disruption and progression	Low levels linked to poor prognosis	Not established	Not yet explored	Biomarker of low-risk disease
**Galectin-3**	Upregulated in epithelium and lymph nodes; cytoplasmic and nuclear	Regulates apoptosis, adhesion, EMT, stemness, immune evasion	Drives metastasis, chemoresistance, and stem-like phenotype	Associated with tumor aggressiveness and poor outcomes	Induces chemoresistance via survival and Wnt signaling	Target for antiinvasive and immune-sensitizing therapy	Marker of invasion, metastasis, and resistance
**Galectin-4**	Overexpressed in poorly differentiated and metastatic GC	Stabilizes c-MET and CD44 signaling; modulates glycosylation	Promotes EMT, receptor activation, peritoneal dissemination	Associated with metastatic potential and poor prognosis	Not defined	Therapeutic target in peritoneal GC	Marker of aggressive behavior
**Galectin-7**	Silenced by promoter methylation	Induces apoptosis, maintains epithelial homeostasis	Tumor suppressor role; inhibits invasion and migration	Downregulation associated with tumor progression	Not reported	Candidate for epigenetic reactivation	Early-stage diagnostic marker
**Galectin-8**	Elevated in non-metastatic GC	Regulates adhesion, integrin signaling, and mTOR stress pathways	Suppresses proliferation and recurrence	Favorable prognostic factor in early GC	Not described	Emerging immunoregulatory target	Marker of low recurrence risk
**Galectin-9**	Context-dependent; elevated in less aggressive tumors	Modulates TIM-3 immune checkpoints, apoptosis, EMT	Dual effect: suppresses spread but enables immune escape	Predicts better survival; upregulated post-therapy	Contributes to adaptive immune resistance	Immunotherapy target (TIM-3/Gal-9 axis)	Predictive biomarker for immune modulation

**Note**: CAFs—cancer-associated fibroblasts; EMT—epithelial–mesenchymal transition; PI3K—phosphoinositide 3-kinase; AKT—protein kinase B; MAPK—mitogen-activated protein kinase; TIM-3—T-cell immunoglobulin and mucin-domain containing-3; mTOR—mechanistic target of rapamycin.

## Data Availability

As a review article, this manuscript does not present original research data. Rather, it provides a synthesis and critical analysis of previously published findings. All data and materials referenced herein are appropriately cited and are available in the cited literature. Accordingly, a data availability statement is not applicable.

## Abbreviations

The following abbreviations are used in this manuscript:

AKT	Protein KinaseB
ANXA2	AnnexinA2
AP-1	Activator Protein-1
α-SMA	Alpha–Smooth-Muscle Actin
Bcl-2	B-cell Lymphoma2
CAF	Cancer-Associated Fibroblast
c-JUN	Jun Proto-Oncogene
c-MYC	Cellular Myc Oncogene
CD4	Cluster of Differentiation4
CD8	Cluster of Differentiation8
CD40	Cluster of Differentiation40
CD80	Cluster of Differentiation80
CDK1	Cyclin-Dependent Kinase1
CDK4	Cyclin-Dependent Kinase4
CXCL12	C-X-C Motif Chemokine Ligand12
CXCR4	C-X-C Motif Chemokine Receptor4
CRD	Carbohydrate-Recognition Domain
CSC	Cancer Stem Cell
DC	Dendritic Cell
ECM	Extracellular Matrix
EMT	Epithelial–Mesenchymal Transition
ERK	Extracellular Signal-Regulated Kinase
FAK	Focal Adhesion Kinase
FOXP3	Forkhead BoxP3
Gal-1	Galectin-1
Gal-2	Galectin-2
Gal-3	Galectin-3
Gal-4	Galectin-4
Gal-7	Galectin-7
Gal-8	Galectin-8
Gal-9	Galectin-9
GC	Gastric Cancer
GLI1	Glioma-Associated Oncogene Homolog1
GM1	Monosialotetrahexosyl-ganglioside1
GRP78	Glucose-Regulated Protein78
GSK-3β	Glycogen Synthase Kinase-3Beta
H-RAS	Harvey Rat-Sarcoma Viral Oncogene Homolog
HIF-1	Hypoxia-Inducible Factor-1
HLA-DR	Human Leucocyte Antigen, DR-Isotype
HMMR	Hyaluronan-Mediated Motility Receptor (CD168)
HER2	Human Epidermal Growth-Factor Receptor2
hTERT	Human Telomerase Reverse Transcriptase
IGF-1R	Insulin-Like Growth-Factor-1 Receptor
IFN-γ	Interferon-Gamma
IL	Interleukin
IM	Intestinal Metaplasia
JNK	c-Jun N-Terminal Kinase
LNM	Lymph-Node Metastasis
MAPK	Mitogen-Activated Protein Kinase
MEK	Mitogen-Activated Protein Kinase Kinase
miRNA	microRNA
mTOR	Mechanistic Target Of Rapamycin
MMP	Matrix Metalloproteinase
MMP-1	Matrix Metalloproteinase-1
MUC1	Mucin1
MUC5AC	Mucin5AC
NCAPG	Non-SMC CondensinI Complex SubunitG
NEO1	Neogenin-1
NF-κB	Nuclear-Factor κ-Light-Chain-Enhancer of ActivatedB cells
NRP-1	Neuropilin-1
PAR-1	Protease-Activated Receptor-1
PC	Peritoneal Carcinomatosis
PD-1	Programmed Cell-Death Protein1
PD-L1	Programmed Death-Ligand1
PI3K	Phosphatidylinositol-3-Kinase
RAS	Rat-Sarcoma Viral Oncogene Homolog
RAF	Rapidly Accelerated Fibrosarcoma Kinase
Rb	Retinoblastoma Protein
ROCK1	Rho-Associated Coiled-Coil-Containing Protein Kinase1
SHP2	Src-Homology-2-Containing Protein-Tyrosine Phosphatase2
Skp2	S-Phase Kinase-Associated Protein2
SMAD2	Mothers-Against-Decapentaplegic Homolog2
SMC	Structural Maintenance of Chromosomes
SNP	Single Nucleotide Polymorphism
SRCC	Signet-Ring Cell Carcinoma
STAT	Signal Transducer and Activator of Transcription
TCF-4	T-Cell Factor-4
TGF-β	Transforming Growth Factor-Beta
Th17	T-Helper-17 Lymphocyte
TIM-3	T-Cell Immunoglobulin and Mucin-Domain-Containing-3
TME	Tumor Micro-Environment
TNM	Tumor, Node, Metastasis (staging)
VCAM-1	Vascular Cell-Adhesion Molecule1
VEGF	Vascular Endothelial Growth Factor
VM	Vasculogenic Mimicry
Wee1	WEE1 G2/M Check-Point Kinase
Wnt	Wingless/Integrated Signaling Pathway
XIAP	X-Linked Inhibitor of Apoptosis Protein
XAF-1	XIAP-Associated Factor1
YAP1	Yes-Associated Protein1
